# Clinical Characteristics and Etiology of Epilepsy in Children Aged Below Two Years: Perspective From a Tertiary Childcare Hospital in South Punjab, Pakistan

**DOI:** 10.7759/cureus.23854

**Published:** 2022-04-05

**Authors:** Zia Ur Rehman

**Affiliations:** 1 Pediatric Neurology, The Children's Hospital and Institute of Child Health, Multan, PAK

**Keywords:** microcephaly, hypotonia, developmental delay, electroencephalography, epilepsy

## Abstract

Background

Epilepsy is described as an enduring disposition toward recurrent unprovoked seizures and by the neurobiological, cognitive, psychological, and social consequences of this condition. This study aimed to find the clinical characteristics and etiology of epilepsy in children aged below two years.

Methodology

This cross-sectional study was conducted at the department of pediatric neurology, the Children’s Hospital and Institute of Child Health, Multan, Pakistan, from February 2021 to July 2021. During the study period, a total of 226 children of both genders, aged below two years, presenting with epilepsy and who underwent electroencephalography (EEG) were included. Socio-demographic and clinical data along with clinical features and radiological/imaging findings were noted.

Results

In a total of 226 children, 121 (53.5%) were male and 105 (46.5%) female. Overall, the mean age was calculated to be 14.6±5.2 months while 107 (47.3%) children were aged between 13 to 24 months. Residential status was found to be rural in 142 (62.8%) children. Generalized seizures (both primary and secondary) were reported in 205 (90.7%) children while the remaining 21 (9.3%) children had focal seizures. The most common etiology of epilepsy was noted to be structural/metabolic in 122 (54.0%) children. Abnormal EEG findings were observed among 150 (66.4%) children. Developmental delay (p=0.0016), hypotonia (p<0.0001), microcephaly or macrocephaly (p<0.0001), abnormal brain CT or MRI (p<0.0001), and abnormal EEG findings (p=0.0161) were found to have a significant association with etiology of epilepsy.

Conclusion

Generalized seizures like tonic-clonic and clonic types were the most common findings among children below two years of age with epilepsy. Structural abnormalities were the most common etiology in children with epilepsy. Age between one to two years was the commonest age of onset of seizures among young children.

## Introduction

Epilepsy is described as an enduring disposition towards recurrent unprovoked seizures and by the neurobiological, cognitive, psychological, and social consequences of this condition [1]. Globally, around 65 million people are estimated to be affected by epilepsy [2]. Recent data stated that 0.6% of children aged below 17 years have active epileptic disorders [3,4]. Epidemiological data from Pakistan estimates a very high prevalence of epilepsy (1%) [5]. Epilepsy is calculated to have a 1% burden in global diseases whereas 80% of epilepsy cases hail from developing nations [6]. Marked differences exist between developed and developing countries in the treatment of epilepsy regarding primary prevention, recognition of the seizures, and accessibility to appropriate and timely treatment options.

Epileptiologists describe the etiology of seizures to be broadly structural/metabolic, genetic, or unknown. These etiologies of epilepsy are based upon the recognized underlying causes of epilepsy which are considered to be very important in terms of clinical evaluation, treatment, and prognostic considerations [7]. Hypoxic-ischemic encephalopathy is one of the major mechanisms behind brain insult that mainly contributes to structural/metabolic seizures. Genetic etiologies of epilepsy are generally because of specific errors like “autosomal dominant nocturnal frontal lobe epilepsy” [8]. During the initial years of life, it might be challenging for clinicians to identify the exact etiology of epilepsy among children [9]. The findings of this study describe the most frequent clinical characteristics and etiological patterns among young children under two years of age presenting at one of the largest tertiary childcare hospitals in South Punjab, Pakistan.

## Materials and methods

Study design, place, and duration of the study

This cross-sectional study was conducted at the department of pediatric neurology, the Children’s Hospital and Institute of Child Health, Multan, Pakistan from February 2021 to July 2021.

Inclusion and exclusion criteria

Children of both genders, aged below two years, presenting with epilepsy and who underwent electroencephalography (EEG) were included. Children with typical simple febrile seizures (single seizure episode) or those presenting with a single provoked seizure were excluded.

Data collection

Approval from Institutional Ethics Committee was taken (Letter No.: CHC/ERC/2021/117). Written and verbal consent were obtained from parents/guardians/caregivers of all patients. During the study period, a total of 226 cases were enrolled as per inclusion/exclusion criteria. Epilepsy was diagnosed as two or more unprovoked seizures that occurred at least 24 hours apart or at least one unprovoked seizure with the probability of recurrence above 60% [10]. At the time of enrollment, socio-demographic and clinical data like gender, age, residential status, family history of epilepsy, clinical presentation, and types of seizures were recorded. Neurological examination was performed by an experienced pediatric neurologist with a post-fellowship experience above three years. Information about developmental milestones, developmental delay, impaired gross motor, fine motor, cognitive, language, and social domains was noted. Clinical features like microcephaly, macrocephaly, dysmorphic features, and abnormal tone or signs of upper motor neuron lesions were also recorded. Baseline investigations including complete blood count, blood glucose, calcium, magnesium, urea, electrolytes, liver function tests, cerebrospinal fluid (CSF) analysis, and blood cultures were asked as needed. Genetic testing including chromosomal and mutation studies was advised when indicated. Radiological studies like cranial ultrasonography, computed tomography (CT), and magnetic resonance imaging (MRI) were performed where considered necessary. All patients underwent an electroencephalogram. Based upon International League Against Epilepsy (ILAE) official report, the electroencephalogram findings along with clinical characteristics were considered to diagnose the etiology of epilepsy. A special proforma was made to record all study data.

Statistical analysis

All the study data were entered and analyzed with the help of SPSS Statistics v. 26.0 (IBM Corp., Armonk, NY). Qualitative data were represented as mean and standard deviation while quantitative variables were shown as mean and standard deviation (SD). A chi-square test was employed to find out the association of various socio-demographic and clinical characteristics of patients with the types of epilepsy. P-value < 0.05 was taken as significant.

## Results

In a total of 226 children, 121 (53.5%) were male. Overall, the mean age was calculated to be 14.6±5.2 months while 107 (47.3%) children were aged between 13 to 24 months. Residential status was found to be rural in 142 (62.8%) children. Generalized seizures (both primary and secondary generalized seizures included) were reported in 205 (90.7%) children while the remaining 21 (9.3%) children had focal seizures. The most common etiology of epilepsy was noted to be structural in 83 (36.1%) children. Abnormal EEG findings were observed among 150 (66.4%) children. Table 1 shows the baseline characteristic of the children with epilepsy in this study.

**Table 1:** Characteristics of Children With Epilepsy (n=226)

Characteristics		Number (%)
Gender	Male	121 (53.5%)
	Female	105 (46.5%)
Age Groups (months)	<1	18 (8.0%)
	1 to 6	42 (18.6%)
	7 to 12	59 (26.1%)
	13 to 24	107 (47.3%)
Residential Status	Rural	142 (62.8%)
	Urban	84 (37.2%)
Types of Seizure	Focal Seizures	21 (9.3%)
	Generalized Seizures	205 (90.7%)
Etiology of Epilepsy	Structural	83 (36.1%)
	Metabolic	39 (17.3%)
	Genetic	36 (15.9%)
	Idiopathic	68 (30.1%)
Developmental Delay		34 (15.0%)
Hypotonia		28 (12.4%)
Microcephaly or Macrocephaly		27 (11.9%)
Abnormal Brain CT or MRI Findings		86 (38.1%)
Abnormal EEG Findings		150 (66.4%)

No statistically significant difference was noted in terms of gender (p=0.6812), age groups (p=0.9844), residential status (p=0.8110), and types of seizures (p=0.8768) with respect to etiology of epilepsy, but developmental delay (p=0.0016), hypotonia (p<0.0001), microcephaly or macrocephaly (p<0.0001), abnormal brain CT or MRI (p<0.0001) and abnormal EEG findings (p=0.0161) were found to have a significant association with etiology of epilepsy. Table 2 shows the distribution of socio-demographic and clinical characteristics of patients with respect to the etiology of epilepsy.

**Table 2:** Distribution of Socio-demographic and Clinical Characteristics of Patients With Respect to Etiology of Epilepsy (n=226)

Characteristics		Etiology of Epilepsy				
		Structural (n=83)	Metabolic (n=39)	Genetic (n=36)	Idiopathic (n=68)	P-Value
Gender	Male	43 (51.8%)	24 (61.5%)	20 (55.6%)	34 (50.0%)	0.6812
	Female	40 (48.2%)	15 (38.5%)	16 (44.4%)	34 (50.0%)	
Age Groups (months)	<1	6 (7.2%)	2 (5.1%)	3 (8.3%)	7 (10.3%)	0.9844
	1 to 6	18 (21.7%)	8 (20.5%)	6 (16.7%)	10 (14.7%)	
	7 to 12	21 (25.3%)	11 (28.2%)	10 (27.8%)	17 (25.0%)	
	13 to 24	38 (45.8%)	18 (46.1%)	17 (47.2%)	34 (50.0%)	
Residential Status	Rural	55 (66.3%)	25 (64.1%)	22 (61.1%)	40 (58.8%)	0.8110
	Urban	28 (33.7%)	14 (35.9%)	14 (38.9%)	28 (41.2%)	
Types of Seizure	Focal Seizures	6 (7.2%)	4 (10.3%)	4 (11.1%)	7 (10.3%)	0.8768
	Generalized Seizures	77 (92.8%)	35 (89.7%)	32 (88.9%)	61 (89.7%)	
Developmental Delay		15 (18.1%)	10 (25.6%)	8 (22.2%)	1 (1.5%)	0.0016
Hypotonia		10 (12.0)	18 (46.2%)	-	-	<0.0001
Microcephaly or Macrocephaly		14 (16.9%)	12 (30.8%)	-	1 (1.5%)	<0.0001
Abnormal Brain CT or MRI Findings		70 (84.3%)	12 (30.8%)	4 (11.1%)	-	<0.0001
Abnormal EEG Findings		49 (59.0%)	26 (66.7%)	20 (55.6%)	55 (80.9%)	0.0161

Children with generalized seizures were further compared for different types of etiologies and details are shown in Table 3. Tonic-clonic types were noted among 123/205 (60.0%) children while 30/205 (14.6%) children had clonic types. It was noted that no significant association (p=0.0784) of types of generalized seizures was revealed with respect to different kinds of etiologies.

**Table 3:** Comparison of Types of Generalized Seizures With Respect to Etiology of Epilepsy (n=205)

Types of Generalized Seizures	Etiology				P-Value
	Structural (n=77)	Metabolic (n=35)	Genetic (n=32)	Idiopathic (n=61)	
Tonic	2 (2.6%)	2 (5.7%)	-	-	0.0784
Atonic	11 (14.3%)	6 (17.1%)	1 (3.1%)	6 (9.8%)	
Clonic	5 (6.5%)	7 (20.0%)	5 (15.6%)	13 (21.3%)	
Tonic-Clonic	53 (68.8%)	16 (45.7%)	22 (68.8%)	32 (52.5%)	
Myoclonic	6 (7.8%)	4 (11.4%)	4 (12.5%)	10 (16.4%)	

## Discussion

As the majority of pediatric epilepsy cases are reported from developing countries, the high prevalence of epilepsy reported is credited to preventable factors like substandard perinatal care, endemic infectious illnesses, and head injuries [11-13]. Factors like inappropriate or delayed diagnosis and treatment along with cultural and social beliefs are some of the other contributing factors to the high prevalence of epilepsy in developing countries [11-13].

In this study, it was found that 53.5% of children reporting epilepsy were male. This is in accordance with the data from another developing African country where 61.0% of children with epilepsy coming to a pediatric neurology clinic were male [14]. As in this study, the majority of the children (62.8%) children belonged to rural areas of residence [14]; cultural and social beliefs might be the reason behind this male predominance among children with epilepsy who are brought to healthcare centers for treatment.

In this study, the mean age was calculated to be 14.6±5.2 months. A recent study from Saudi Arabia evaluating children below two years of age with epilepsy found the mean age of the children to be 16 months, which is quite close to what we noted [15]. A study by Alonazi et al. observed that 7.9% of children with epilepsy have focal seizures [16] while in this study, it was seen that 9.3% of children with epilepsy had focal seizures. We also reported generalized seizures including tonic-clonic seizures to be the commonest types of generalized seizures. Our findings are very similar to what was documented by Khreisat et al. [17]. As these types are described as the preventable types of generalized epilepsy, parents/caregivers or treating physicians might be missing the initial onset of seizures which might have initiated as a focal type but could have progressed into generalized forms [18]. A study from North America found different findings in terms of types of seizures as they reported etiology being structural or metabolic in 33% of the patients; this contrasts with our findings and could be due to differences in epidemiological aspects of epilepsy found in different parts of the world [19].

No identifiable causes of epilepsy were found in 30.1% of children. A study from Saudi Arabia reported that 44% of children with epilepsy have idiopathic epilepsy which is what is reported in this study [16]. Another study found that only 20% of the patients have idiopathic epilepsy [17]. We identified that 15.9% of children in this study had epilepsy with a genetic etiology. Moreover, other researchers in the past have pointed around 13% of cases of epilepsy have genetic etiology [16,20]. Recent decades have seen significant advancements regarding the identification of the genetic etiology of epilepsy [21].

Limitations of the study

Being a single-center study conducted in a childcare setting in South Punjab, Pakistan, our findings should not be generalized. We were unable to record treatment outcomes among the current set of patients.

## Conclusions

Generalized seizures like tonic-clonic and clonic types were the most common findings among children below two years of age with epilepsy. The age between one to two years was the commonest age of onset of seizures among young children. Structural abnormalities were the most common etiology in children with epilepsy.
